# Talking to the people that really matter about their participation in pandemic clinical research: A qualitative study in four European countries

**DOI:** 10.1111/hex.12634

**Published:** 2017-09-27

**Authors:** Nina H. Gobat, Micaela Gal, Christopher C. Butler, Steve A.R. Webb, Nicholas A. Francis, Helen Stanton, Sibyl Anthierens, Hilde Bastiaens, Maciek Godycki‐ćwirko, Anna Kowalczyk, Mariona Pons‐Vigués, Enriqueta Pujol‐Ribera, Anna Berenguera, Angela Watkins, Prasanth Sukumar, Ronald G. Moore, Kerenza Hood, Alistair Nichol

**Affiliations:** ^1^ Division of Population Medicine School of Medicine Cardiff University Cardiff UK; ^2^ Nuffield department of Primary Care Health Sciences Medical School Division Oxford University Cardiff UK; ^3^ University of Western Australia Cardiff UK; ^4^ Department of Primary and Interdisciplinary care Faculty of Medicine and Health Sciences University of Antwerp Wilrijik Belgium; ^5^ Centre for Family and Community Medicine Faculty of Medical Sciences Medical University of Lodz Lodz Poland; ^6^ Institut Universitari d'Investigació en Atenció Primària Jordi Gol (IDIAP Jordi Gol) Barcelona Spain; ^7^ University College Dublin School of Medicine Dublin Ireland; ^8^ Centre for Trials Research Cardiff University Cardiff UK; ^9^ HRB funded Irish Critical Care‐Clinical Research Core University College Dublin School of Medicine Dublin Ireland

**Keywords:** epidemic, infectious disease outbreak, influenza, informed consent, pandemic, patient and public involvement

## Abstract

**Background:**

Pandemics of new and emerging infectious diseases are unpredictable, recurrent events that rapidly threaten global health and security. We aimed to identify public views regarding provision of information and consent to participate in primary and critical care clinical research during a future influenza‐like illness pandemic.

**Methods:**

Descriptive‐interpretive qualitative study, using focus groups (n = 10) and semi‐structured interviews (n = 16), with 80 members of the public (>18 years) in Belgium, Spain, Poland and the UK. Local qualitative researchers followed a scenario‐based topic guide to collect data. Data were transcribed verbatim, translated into English and subject to framework analysis.

**Results:**

Public understandings of pandemics were shaped by personal factors (illness during the previous H1N1 pandemic, experience of life‐threatening illness) and social factors (historical references, media, public health information). Informants appreciated safeguards provided by ethically robust research procedures, but current enrolment procedures were seen as a barrier. They proposed simplified enrolment processes for higher risk research and consent waiver for certain types of low‐risk research. Decision making about research participation was influenced by contextual, research and personal factors. Informants generally either carefully weighed up various approaches to research participation or responded instinctively. They supported the principle of using routinely collected, anonymized clinical biological samples for research without explicit consent, but regarded this as less acceptable if researchers were motivated primarily by commercial gain.

**Conclusions:**

This bottom‐up approach to ascertaining public views on pandemic clinical research has identified support for more proportionate research protection procedures for publically funded, low‐risk studies.

## 1 INTRODUCTION

Pandemics and epidemics of new and re‐emerging infectious disease are unpredictable but recurrent events that threaten global health and socio‐economic security. It is of utmost importance to human health that there is capacity to conduct time‐critical, patient‐centred research during a pandemic because such research offers the best strategy for mitigating impact on health and society.^1^, ^2^, ^3^ Such research is necessary to help clinicians accurately diagnose and provide optimal treatment, to develop preventive interventions such as vaccines, and for policymakers to make decisions about strategies to prevent spread and mobilize health‐care services. One of the major conclusions from the, fortuitously mild, 2009 H1N1 pandemic is that globally co‐ordinated research capacity that can be rapidly mobilized is lacking.

Globally, the call has been made for pandemic planning to incorporate preparedness and capacity for conducting prospective patient‐focused clinical research.^2^, ^4^ Such research should be relevant, methodologically rigorous and be held to the same standards of ethical conduct as research conducted in non‐emergent times.^5^ However, there are numerous barriers to conducting clinical studies during a public health emergency, not least of which is ensuring that participants have sufficient information and opportunity in which to make valid, informed decision about research participation. Routine research enrolment processes, which are both appropriate and important under usual circumstances, might be both disproportionate and counterproductive during a serious pandemic. The potential threat to human health that would occur with a serious pandemic is of such a magnitude that we must examine all barriers that prevent us conducting effective clinical research. An important but seldom examined consideration, when balancing the rights and protection of research participants against the public health impact of research, is the views and opinions of prospective research participants themselves.

The objective of this study was to ascertain the opinions of members of the public in four European countries regarding provision of information and consent to participate in research that should apply during a pandemic. Specifically, we sought to describe public understandings of pandemics and related research, to identify factors that influence people's decisions regarding research participation and to consider acceptability of alternate methods of enrolment to clinical research during a pandemic.

## 2 METHODS

### 2.1 Design and setting

This study was conducted as part of the European Commission funded Platform foR European Preparedness Against (Re‐)emerging Epidemics consortium (PREPARE; www.prepare.eu). It aims to inform study optimization of multisite, pan European clinical studies in primary and critical care for over 5000 patients during interpandemic periods and in preparedness for a research response during a future pandemic. We used qualitative research methodology because we aimed to identify important issues from the perspectives of the public before attempting to quantify them. We therefore prioritized quality and richness of information gathering over quantity or prevalence of views. This work has informed construction of a survey instrument that is being applied to a large, representative sample.

### 2.2 Recruitment and sampling

We selected one country each from northern, southern, eastern and western Europe, as defined by the United Nations macro‐geographical regions.^6^ Focus groups and interviews took place between July and November 2015 in Cardiff, UK; Barcelona, Spain; Lodz, Poland; Antwerp and Bruges, Belgium. These locations also had teams that were members of the PREPARE consortium recruiting patients into pandemic research. Additionally, their health services differ in important, contrasting ways (eg fee for service and free at the point of delivery). Local, experienced qualitative researchers with good command of the English language were recruited through pre‐existing research networks. Informants (>18 years) within the four countries were purposively sampled to include parents, carers and informants with experience of hospital admission. Participant recruitment was by local advertisement. Interested respondents completed a short demographic questionnaire before being invited to attend the group.

### 2.3 Data collection

Focus groups and semi‐structured individual interviews were used. Our rationale for using focus groups was to stimulate discussion between those involved, anticipating that this would give rise to varied ideas and perspectives beyond those of the research team.^7^, ^8^ We held 10 two‐hour focus groups. Topic guides were developed iteratively in two pilot focus groups (UK). Two focus groups were subsequently held in each of the four countries in accessible community venues. Pilot data were included in the data corpus as there were no substantive changes to data collection procedures that invalidated their use. We conducted 16 follow‐up telephone interviews (15‐30 minutes in length) within 2‐7 days of focus groups with two informants from each group. We selected interviewees for these individual interviews who were less vocal in the group or those who had held discrepant or contrasting views.^7^ Focus groups and interviews were conducted in the local language. A face‐to‐face training meeting of all focus group moderators was held prior to data collection to ensure consistency, cultural sensitivity and to inform reflexivity. All study materials were translated into local languages. Focus groups were conducted in the local language by two local researchers (one moderator and one observer). Local researchers came from a range of different backgrounds (sociology, primary care, public health, culture science). Post‐group debriefing and the use of field notes further enhanced reflexivity.

Focus group questions were rooted in evidence on pandemic clinical research and standards for good clinical practice in research.^4^, ^9^, ^10^ We used images and hypothetical scenarios of a moderate‐risk influenza pandemic to stimulate discussion of participating in the following three research scenarios: a point of care test evaluation (primary care), evaluation of a routinely used antiviral medication with known safety profile (primary care) and evaluation of a newer antiviral medication with known safety profile (hospital intensive care unit) (Box 1). We also asked about the acceptability of conducting research that uses excess from routinely collected clinical samples, and enrolment to adaptive clinical trials (Box 1). We selected a moderate‐risk pandemic influenza because an airborne virus, such as a novel influenza, is a likely candidate for an epidemic or pandemic spread that might affect European countries. Scenarios were aligned with clinical studies being conducted in PREPARE that aim to recruit patients from primary care and critical illness settings and that aim to generate evidence to inform clinical management of outbreak‐related illness. We chose to avoid an outbreak with very high mortality, such as Ebola, which might polarize decisions and limit transferability of findings to a European context. We also asked about reasonable adjustments to research enrolment processes, that is from the point when a patient is identified as eligible to be invited into a study, through the process of information exchange to support their decision about participation, and, if selected, the provision of consent. Follow‐up interviews aimed to capture post‐group reflections.

### 2.4 Analysis

Focus groups and interviews were audio‐recorded, transcribed verbatim and translated into English. Local researchers checked each stage for accuracy. Data were analysed using the framework approach, which follows a systematic process moving from data management to description to interpretation.^11^ We charted the focus group (NG) and interview (HS) data by identifying text that related to our study objectives as evidence of a particular theme. In this way, a visual overview of the data was produced from which patterns, including similarities and differences, could be identified. In practice, this involved charting data related to each specific objective on multiple Excel spreadsheets, which then formed the basis for team discussions regarding emergent, explanatory themes. Charting the data enabled recognition of contradictory or opposing views. The process of charting gave rise to the initial coding frame, which was then sent to local researchers (MG, SA, MPV), who double coded one of their focus group transcripts, and gave feedback on the analytic frame. At this point, the analytic process moved further into an interpretative stage where links between themes and cases were explored to expand the explanatory frame. In this higher‐level analysis, we aimed to identify the range and diversity of factors influencing decision making and retrospectively applied decision‐making theory^12^ in our analyses. NVivo 10 software (QSR International, Melbourne, Australia) was used to facilitate analysis.

### 2.5 Rigour and quality criteria

We used the COREQ checklist when designing and reporting this study^13^ and adhered to the following rigour criteria^14^: description of context, of informants and of the research process, methodological adequacy and reflexivity of the multidisciplinary research team. Throughout the research process, we actively sought opportunities to consider how multiple perspectives influenced the research process.

### 2.6 Ethical considerations

Researchers obtained local ethical approvals in each country. Informants gave voluntary, written informed consent, and researchers guaranteed anonymity, confidentiality and data protection.

## 3 RESULTS

Table 1 reports the characteristics of the 80 informants. Results were generally consistent across countries unless otherwise indicated in the text. In our follow‐up interviews, no informants felt they had been misrepresented or had revised their opinions.

**Table 1:** Characteristics of focus group informants

	Belgium (n = 16)	Spain (n = 16)	Poland (n = 19)	UK (n = 29)^a^
Gender				
Female	10	11	11	15
Male	6	5	8	14
Age				
Median (Interquartile range)	60‐69 (40‐69)	50‐59 (30‐70)	60‐69 (50‐69)	30‐39 (30‐49)
18‐29	0	0	2	7
30‐39	1	5	2	8
40‐49	5	2	0	7
50‐59	1	3	4	3
60‐69	7	1	10	4
>70	2	5	1	0
Nationality				
Local (Belgian, Spanish, Polish, British)	16	14	19	27
Other	0	2	0	2
Parent or carer				
Yes	11	3	12	17
No	5	13	7	12
Intensive care unit experience				
Yes	1	1	‐	6
No	11	15	‐	23
Unsure	4	‐	19	‐

a We included data from two pilot focus groups held in the UK.

### 3.1 Public understanding of pandemics

Informants described pandemics using both rational and emotive explanations. Rational descriptions included the origin, characterization, transmission, spread and consequences of a pandemic.

P3 …somewhere in Thailand a zoonotic virus mutates and infects 100 million people. (Poland)

P6 … half of the working population is no longer working because of.. whatever the long‐term effect is. (Belgium)

Emotive descriptions included the unpredictability of an outbreak, the rapidity of spread, high mortality and difficulty with containment. Consequently, they described fear, crisis and panic as defining features of a pandemic.

P5 Well, everyone was worried about Ebola, not because there was one case, but because **it could have gone out of control** and we didn't know how to tackle it, or we didn't have the systems to fight against it. (Spain)

P9 …people die, there is no escape. (Poland)

Personal (eg illness and vaccination experience) and social factors (eg history, media, political drivers) shaped participant understandings and perceptions of pandemics. In Belgium, Spain and the UK, but not Poland, the media was described as sensationalizing and stereotyping pandemic outbreaks, resulting in potential desensitization to the real threat of a future outbreak.

P1 …. there's so much panic in a lot of the press anyway especially in [name of paper] and [name of paper] that you become a little bit desensitised to everything … so when a new pandemic comes around you think, ‘okay swine flu didn't really do that much to me’ (Wales)

P3 But afterwards it doesn't turn out to be nearly as bad as expected, all of it. A huge fuss is made about it in the press and in the end, OK well a lot of people were a bit sick (Belgium)

Informants referred to historical pandemics such as the Black Death, Cholera in London, Spanish flu and, more recently, Ebola. Awareness of these outbreaks contributed to a perception that pandemics, by definition, are highly infectious, spread rapidly and result in high mortality. In contrast, the 2009 swine flu (“H1N1”) pandemic was associated with expectations of milder illness. Public health information distributed through official channels was seen as trustworthy and as able to contribute to a more balanced understanding of what pandemics involve. However, informants described particular news stories or events as having introduced new perspectives. For example, the social activism of a Catalan physician during the swine flu epidemic, who heavily criticised the pharmaceutical industry, was cited as a reason to be cautious about the motives of those conducting research. Informants also noted criticisms of public health agencies and in one group, in Wales, informants reflected on the political dimension to pandemics, where political parties are under pressure to respond effectively to pandemic outbreaks.

### 3.2 Attitudes towards pandemic research and willingness to participate

Informants regarded pandemic research as valuable and potentially life‐saving. In identifying research priorities, informants highlighted the potential impact of research outcomes on their own lives. They wanted effective treatment, accurate diagnosis and targeted and public health information for example, on risks of infection, health protection and symptom recognition. Consequently, they saw value in research to understand the causes, route of transmission, clinical manifestation, disease course and to identify at‐risk groups. Informants also saw value in disease surveillance programmes and for research plans to be in place in anticipation of a future outbreak.

P10 (once the outbreak has started) it would be then too late for research (Poland)

Informants were generally willing to take part in the research scenarios we presented. However, they saw existing enrolment procedures as a barrier to their participation, due to the time required and the cognitive burden on patients when they were unwell or on relatives when their loved ones were unwell (eg in acute illness settings). Some informants felt enrolment processes created suspicion and questioned the validity of prospective informed consent when they or members of their family were unwell or distressed.

P2 I'm thinking if you're feeling that rough, you're not thinking straight anyway so wrong decisions could be made (UK)

In Belgium, Spain and the UK, informants described desensitization to authorization processes as a consequence of being inundated with signing “terms and conditions” in daily life. As a result, authorization has become habitual and automatic and has lost meaning.

P6 … you sign things every day, …: you agree to the terms and condition …. Agreed? Bang. Do you know what you've signed? You've agreed…

P5/P4 That's quite right, you sign lots of things…

P6 And then for scientific research you're going to say: I have to sign all this, I don't think it's so bad to have to sign it. (Belgium)

A minority of informants felt that current regulations protected clinicians and researchers rather than research informants. However, on the whole, informants appreciated the protections afforded by research regulation and generally saw these as reassuring, professional and indicative that research was trustworthy.

P8 I think these consents, they are meant to cover the doctor's back, not yours (Spain).

Informants saw pandemics as an exceptional circumstance and felt that different rules or thresholds for pandemic research should apply. They proposed abbreviated information sheets and consent forms and suggested alternative ways of exchanging information (pictures and brief videos) and taking consent (verbal or video consent taken by clinician). The need for accountability and a verifiable “paper trail” was factors influencing unacceptability particularly of verbal consent. For low‐risk interventions or where there was little difference between what would happen if they took part in research compared with routine treatment (eg taking an already licensed medication), informants suggested automatic enrolment with opt‐in or opt‐out provision.

P9 I think it should be that everybody is opted in and opt out and that would be the fastest (UK)

P9 On the other hand: if the doctor prescribes something for us, we generally take it without thinking about whether it's really suitable for the particular situation, and we don't sign anything (Belgium).

P9 No, you can't burden someone with such a decision.

P3 There's no need. You can write a declaration of intent, just as you can write a declaration of intent on donating your organs after death. (Poland)

In endorsing these models of research enrolment, informants referred to organ donation initiatives. Thus, in Belgium and the UK (Wales), opt‐out policies for organ donation were referenced. In Poland, parallels with opt‐in to organ donation registers were made. For ICU research, informants recognized the need for a third‐party to consent on their behalf should they lack decision‐making capacity. Preference was expressed for a close family member who could represent their wishes and values to decide. However, if family members were unavailable (for example, ill with the pandemic), some informants, particularly those with prior ICU experience, considered it acceptable for a doctor to decide on their behalf. High levels of trust in health‐care professionals and the relatively low risks of harm with involvement in the study determined acceptability.

### 3.3 Factors influencing decisions about research participation

Decisions about participation were influenced by a complex interplay between personal attributes, research factors and contextual cues. Personal factors, such as age, life stage and previous ICU experience, were influential. For example, older informants felt their age made them more “philosophical” and described feeling more socially conscious, while parents of younger children described needing to be especially cautious about exposing themselves to risk. Research factors, particularly the benefits and risks or burdens of participation, were deliberated. These were judged contextually. For example, when deliberating about participation in the ICU scenario, risks associated with acute illness and the pandemic were considered.

Perception of immediate personal benefit was a strong motivator to take part. For example, a more accurate diagnosis was offered as justification to be part of an evaluation of a point of care test (POCT) in primary care (scenario 1).

P4 If they do this test, regardless how reticent you are… if you can know there and then whether you are infected…

P7 Of course, then yes…

P4 … or whether you aren't infected… I think you should assume it is worth it… (Spain)

Perceptions regarding the benefit of antiviral medication in primary care (scenario 2) were mixed, but some respondents saw benefits to receiving this trial intervention if offered.

P6 f it shortens the period of infection for 1 week… then I would. (Spain)

In the ICU scenario (scenario 3), informants perceived that they would receive more effective treatment if in a trial than if not. Some informants said they would only participate if guaranteed to receive an experimental trial intervention. Fear, driven by a perception that that no known effective treatment may be available for a life‐threatening illness meant that informants placed greater hope in the intervention's superiority. Informants were also motivated by altruism, social responsibility and by contributing to medical knowledge in the “long‐term.” They wanted to receive feedback about the outcome of research they supported and felt feedback of results was a neglected aspect of research participation, which reduced the chances of subsequent research involvement.

Benefits were weighed against perceived risks and burdens of research participation. For example, informants saw a hypothetical POCT requiring a nasal swab sample in primary health care (scenario 1) as minimally invasive and low risk and used phrases such as “*its just a swab*,” “*sounds harmless*” and “*no bother*” as justifications for their decision to take part should they be invited. Risks of side‐effects and scepticism that medication was necessary were reasons given not to take part in the primary care medication study (scenario 2).

P1 … I thought that with a viral infection you just have to let it run its course. …. So I'd have more objections in this case. I think: leave me alone; I'll let it run its course…. (Belgium)

In the ICU scenario (scenario 3), risks and burdens were judged proportionate to the context of critical illness in which the decision about research participation was being made. Informants expressed less concern about potential side‐effects of new interventions, describing the critical illness scenario as being “*serious*” and “*you're in dire straits*” (*Poland*).

However, informants also described making a more instinctive, intuitive decision based on personal pre‐disposition, rapid appraisal and perceptions of trust, particularly when feeling fearful or unwell.

P1 I think that if you're not sick, you definitely think like: would I do it or wouldn't I? Weigh up the pros and cons more. And think about it. And if you're ill, you'd think more radically like: no, leave me alone. Or: yes, do it. (Belgium)

P7 I would usually take part, because this is my way of being (Spain)

In primary care, informants described a high level of trust in clinicians that had developed over time and with continuity of care. Several informants spoke of actively seeking to consult with primary care clinicians they trust; care pathways in Poland and Belgium allow patients to choose their primary care physician and this is possible to some extent in Spain and the UK. Informants with experience of ICU spoke of trust developing very rapidly with treating clinicians by virtue of the intensity of acute illness and vulnerability coupled with perceived expertize of the clinical team, including the quality of their communication. Informants felt they would seek advice from these clinicians about research participation. A clear, minority view was also expressed suggesting that informants may feel unable to refuse participation in the context of an existing relationship with their treating clinician.

### 3.4 Acceptability of using routinely collected clinical samples for research

Informants supported the use of routinely collected clinical samples being available for pandemic research. Altruism was a key motivation and informants considered it prudent and efficient not to waste clinical samples. Informants mostly thought that this should be allowed without specific consent, as long as safeguards were in place to protect against identification of the individuals.

P3 I wouldn't mind.

P8 Yes, I agree, I think it is important to make the most of it because… if it is anonymous, if it is only to do more research, then they shouldn't have to ask for permission again. At the signing stage, you could indicate ‘I sign up to this research and for the following research studies derived from it’. (Spain)

However, a minority of respondents from all countries wanted upfront information about how and for what their sample might be used. They also sought assurance that their samples would not be used for commercial purposes and were generally less trusting of research funded by industry sponsors, or where financial gain was the main ultimate motive. A counter‐argument was also raised, highlighting the need to support investment required to develop new, more effective products.

### 3.5 Views on Participating in Adaptive Clinical Trials

Informants grasped the concept of the response‐adaptive clinical trial design and viewed this as well suited to a pandemic context and liked the novelty, flexibility and potential for enhanced personal benefit.

P1 The second one makes more sense …because you can adjust the adaptive trial. (Belgium)

P7 The other one takes more into account the person. (Spain)

They took a pragmatic view about the potential disadvantage to being enrolled in a response‐adaptive trial at an earlier stage, when less was known about which treatment was performing better.

P2 **you're ill when you're ill** aren't you?

P8 exactly (Wales)

A minority view held that the adaptability of the design implied that researchers were uncertain, “*fiddling in the middle*” (P4, UK 04) and that “*perhaps traditional is safer, it can't go wrong*” (P8, Spain10). On balance, informants felt that being given information about study design was less important than information about risk, burdens and benefits in informing their decision about taking part or not. If study design information was to be available upfront, informants said they would want to know about the potential for adaptation and how that might impact their involvement.

P5 We consent to participate in the study, don't we? And the study techniques, well, this is a matter for the doctor who conducts it. (Poland)

## 4 DISCUSSION

We present a wide range and diversity of public views in four European countries about perceptions of research participation during a pandemic outbreak of a novel Influenza‐like‐illness. Informants supported the need for clinical research during a pandemic outbreak and endorsed simplified, proportionate research enrolment processes. Informants valued regulatory oversight of clinical research but also described having become desensitized to consent and “disclaimer” rituals and would not want to be overburdened with decision making about participation in low‐risk research when they were unwell. They viewed opt‐out consent models for low‐risk research as acceptable and, at times, preferable.

Continued public engagement about clinical research participation is essential, in particular, to clarify the distinction between clinical care and research participation and to mitigate therapeutic misconception.^15^ In a pandemic outbreak, therapeutic misconception may be amplified as agreeing to be part of a trial might be seen as providing unique access to novel or better treatments, rather than access to treatments with, as yet, unknown effectiveness or superiority.^15^ While therapeutic misconception may inadvertently facilitate recruitment into clinical trials in the short term, in the longer term, it undermines trust in science, transparency and good clinical practice. In a pandemic outbreak, it is likely that public health agencies would determine whether promising interventions should be routinely available to the public. Novel therapeutic agents may only be available for testing in a randomized controlled trial if sufficient uncertainty regarding their efficacy remained. Nevertheless, public perception that novel research interventions are superior and therapeutic misconception should be addressed explicitly during recruitment itself. Response‐adaptive trials may be appropriate for assessing certain types of interventions in pandemic research^1^, ^16^ and may go some way to meet therapeutic expectations about trial participation in that these trial designs allow pre‐specified changes to the chances of a participant being allocated to an intervention arm that starts to perform better,^17^ thus participants joining a study later may be more likely to receive an effective intervention.^18^, ^19^ Some have raised concern that such studies can be complex to explain to patients,^20^, ^21^ and so could further complicate enrolment in a public health emergency. Our informants could grasp the response‐adaptive trial design and saw it as advantageous, particularly during a pandemic outbreak, but did not necessarily consider information about study design as a priority during an outbreak. Our findings confirm the general public's acceptance of donating excess material from routinely collected clinical samples for biomedical research provided safeguards are in place to protect against identification of the individuals and that the research was being performed primarily for patent benefit rather than to make a profit.^22^

Decisions about participation in research were influenced by a complex interplay between personal attributes, research factors and contextual cues. In some scenarios, informants deliberated, weighed the risks and benefits of taking part in the context of perceived illness severity and pandemic threat. Informants also described a more instinctive decision‐making style. They described a pre‐disposition to take part in clinical research or not, and then sought cues, particularly regarding trustworthiness, to confirm or contradict that position. Others have made similar observations suggesting that rapid decision making at a time of increased risk and uncertainty may reflect a natural way that people have evolved to competently navigate complex environments.^23^ These insights about the nuances of participant decision making challenge assumptions underpinning our current models of research enrolment and suggest they may be oversimplified. They also highlight the importance of public trust as an important foundation for involvement during a pandemic outbreak. Focus group informants in all countries made reference to national events that had either eroded or enhanced trust in both commercially and publically funded institutions. For example, Poland was the only country not to stockpile the vaccine available during the swine flu pandemic, which our informants saw as prudent. In the UK, informants trusted the nationally funded health service and described an obligation to “give back” wherever possible, thereby justifying research participation.

Through their participation in this study, informants gained insight and knowledge, formulated their position on research participation and spoke with family about research wishes. While informants did not necessarily change their views about research participation, they described increased awareness and valued the opportunity for debate. Other studies have identified similar effects.^24^ People need opportunity to formulate their views on research participation and communicate these with people close to them. National initiatives such as organ donation have stimulated and normalized discussions of people's wishes. Similar campaigns might contribute to a broader public discourse about research participation. Informants in Belgium, Poland and the UK (Wales) all made reference to organ donation. Interestingly, discussion about organ donation was not raised spontaneously in the Spanish groups despite Spain leading the way in opt‐out organ donation. We have developed a public facing report of our findings to invite continued engagement among our study participants and to extend discussion and debate among a wider audience (Box 1).

### 4.1 Strengths and weaknesses

Sample sufficiency was attained with the use of complementary techniques and the richness of data collected across four countries. Richness of data was achieved, in part, by skilful group facilitation through which informants felt comfortable to change their opinions or express discrepant views; researchers actively elicited individual and group deliberation. Similarity of findings in the different countries and with findings from other studies, using follow‐up interviews with participants holding discrepant views and reflexive discussions on analysis among researchers, supports the credibility of our research. Our sample is not reflective of the age group most affected during the H1N1 pandemic; however, we have no way of predicting in advance which age groups will be affected in future pandemics. Our groups were largely mono‐cultural, and the views of minority ethnic groups are not represented. As respondents volunteered to participate, we assume some responder bias. We used hypothetical scenarios and views may have been different had we used different scenarios, or following actual research participation during a pandemic. Process evaluations conducted as part of pandemic clinical research are required to offer insight into participants’ actual experience. Despite training, there was some variation in the way informants were recruited and data were collected in different countries, which may have impacted our ability to compare across countries. The median age differences in different country data also limits our ability to compare directly across countries. Appropriate to the hypothesis generating nature of this work, we worked with a purposive sample which is designed to provide transferrable but not generalizable data.^25^ We have used these findings to develop a cross‐sectional survey of public opinion that will allow us to quantify views and consider generalizability in nationally representative samples in eight countries within the Organisation for Economic Co‐operation and Development (OECD).

## 5 CONCLUSIONS

This work underpins the call to minimize regulatory burden for pandemic clinical research proportionate to the potential risk of harm. Pandemic research requires flexibility, adaptability and co‐ordination and sets an imperative for change in the current paradigm of research. The public value safeguards of research regulation, but these should not present disproportionate, “one‐size‐fits‐all” barriers to their participation. Opportunities for change may lie in new regulations guiding European clinical trials that now make provision for a new category of clinical study that poses minimal risk to participants.^26^ Newly published ethical guidelines also offer advice regarding modifications to informed consent.^27^ Effective pandemic preparation for clinical research requires active public involvement to mitigate therapeutic misconception, engender trust and promote innovation to the research process.

## CONFLICT OF INTERESTS

The authors declare that they have no conflict of interests.

## AUTHORS’ CONTRIBUTIONS

NG co‐led the study design and implementation, obtained ethics approvals, recruited participants, collected data, led data analysis and drafted the manuscript. She is guarantor. MG co‐led the study design and implementation, obtained ethics approvals, recruited participants, collected and double‐coded data, and provided critical review of the manuscript. CCB conceived the idea and contributed to study design, analysis and interpretation. SW contributed to study design, analysis and interpretation. NAF contributed to study design, data collection in Wales and analysis. HS analysed interview data. SA and HB (Belgium), MGo and AK (Poland), MPV, EPR and AB (Spain) implemented the study in their country (obtained ethics approvals, recruited participants, collected data, checked transcriptions and provided critical review of the manuscript). AK also transcribed data (Poland) and SA (Belgium), MPV (Spain) double‐coded data. AW project administered the study, co‐ordinated the focus groups in Wales and co‐ordinated transcription and translation. KH, RM, PS contributed to study design. AN contributed to study design, analysis and interpretation. He leads PREPARE‐EARL and is principal investigator. All authors approved the final manuscript.

## ETHICS APPROVAL AND CONSENT TO PARTICIPATE

Ethical approval was granted by the School of Medicine Research ethics Committee, Cardiff University, Cardiff, Wales, UK; Bioethics Committee of the Medical University of Lodz, Lodz, Poland; Clinical Ethics Committee of the IDIAP Jordi Gol and Gurina, Barcelona, Spain; and Antwerp University Ethics Committee, Antwerp, Belgium.

## CONSENT FOR PUBLICATION

Not applicable.

## AVAILABILITY OF DATA AND MATERIAL

The datasets generated and/or analysed during the current study are not publicly available due to protection of participant confidentiality but are available from the corresponding author on reasonable request.
