# Supplementary material for: Talking to the people that really matter about their participation in pandemic clinical research: A qualitative study in four European countries
Source: Health Expect. 2017 Sep 27;21(1):387–95. doi: 10.1111/hex.12634 (PMC5750735; doi:10.1111/hex.12634)
Supplement: Supplementary file 1 [file HEX-21-387-s001.docx]

Box 1: Scenarios used in focus group discussions

| Pandemic context: used in all scenarios. | *A new pandemic flu-like illness has been causing severe illness in people in Asia over the past 5 months. Cases of people getting sick with this illness are now happening in [country] and within the past few weeks people in [city] have been getting ill, including one of your colleagues.* |
| --- | --- |
| Scenario 1: evaluation of point of care test, primary care | *A new test is available that can be used in GP practices. The test gives results in about 15 minutes and doctors think the results can help them make a more accurate diagnosis. This test needs to be evaluated in everyday clinical practice. Doctors will need to take a swab from the back of a patient’s nose to do the test.* |
| Scenario 2: evaluation of routinely used anti-viral medication, primary care | *You will receive one of two medications. Both have been used previously, are considered safe for use and have similar side effect profiles. Doctors are not sure how well they will work with this new virus. This research will let doctors know which medication works best and for which group of patients.* |
| Scenario 3: evaluation of new anti-viral medication, intensive care | *You will be given Flu z, a new Flu medication that has been clinically tested as safe and likely of benefit to this new infection. Doctors think the new medication cuts down the length of the illness and makes symptoms less severe but it needs to be given as soon as possible after symptoms first appear to be most effective. As part of the research, you will also need to have two extra blood samples (about 2 teaspoons) and a nose swab taken as soon as possible and then again a few days later.* |
| Adaptive clinical trials | *A traditional research study starts and stops at a predetermined time and enrols a certain number patients. In an adaptive design, there are certain ‘stop points’ where data are analysed as the study goes along. The study can then change based on the results. For example, if one treatment appears to be better a larger number of patients would receive the likely ‘ better’ treatment. As a result, the longer the study is being run for, the more likely you would be to receive an effective treatment. So your chance of being allocated to an effective treatment is like the flip of a weighted coin.* |
